# Imaging Atherosclerosis by PET, With Emphasis on the Role of FDG and NaF as Potential Biomarkers for This Disorder

**DOI:** 10.3389/fphys.2020.511391

**Published:** 2020-10-22

**Authors:** Michael Mayer, Austin J. Borja, Emily C. Hancin, Thomas Auslander, Mona-Elisabeth Revheim, Mateen C. Moghbel, Thomas J. Werner, Abass Alavi, Chamith S. Rajapakse

**Affiliations:** ^1^Department of Radiology, Hospital of the University of Pennsylvania, Philadelphia, PA, United States; ^2^Perelman School of Medicine at the University of Pennsylvania, Philadelphia, PA, United States; ^3^Lewis Katz School of Medicine at Temple University, Philadelphia, PA, United States; ^4^Division of Radiology and Nuclear Medicine, Oslo University Hospital, Oslo, Norway; ^5^Institute of Clinical Medicine, Faculty of Medicine, University of Oslo, Oslo, Norway; ^6^Department of Radiology, Massachusetts General Hospital, Boston, MA, United States; ^7^Department of Orthopaedic Surgery, Hospital of the University of Pennsylvania, Philadelphia, PA, United States

**Keywords:** atherosclerosis, molecular imaging, PET, fluorodeoxyglucose, sodium fluoride

## Abstract

Molecular imaging has emerged in the past few decades as a novel means to investigate atherosclerosis. From a pathophysiological perspective, atherosclerosis is characterized by microscopic inflammation and microcalcification that precede the characteristic plaque buildup in arterial walls detected by traditional assessment methods, including anatomic imaging modalities. These processes of inflammation and microcalcification are, therefore, prime targets for molecular detection of atherosclerotic disease burden. Imaging with positron emission tomography/computed tomography (PET/CT) using 18F-fluorodeoxyglucose (FDG) and 18F-sodium fluoride (NaF) can non-invasively assess arterial inflammation and microcalcification, respectively. FDG uptake reflects glucose metabolism, which is particularly increased in atherosclerotic plaques retaining macrophages and undergoing hypoxic stress. By contrast, NaF uptake reflects the exchange of hydroxyl groups of hydroxyapatite crystals for fluoride producing fluorapatite, a key biochemical step in calcification of atherosclerotic plaque. Here we review the existing literature on FDG and NaF imaging and their respective values in investigating the progression of atherosclerotic disease. Based on the large volume of data that have been introduced to the literature and discussed in this review, it is clear that PET imaging will have a major role to play in assessing atherosclerosis in the major and coronary arteries. However, it is difficult to draw definitive conclusions on the potential role of FDG in investigating atherosclerosis given the vast number of studies with different designs, image acquisition methods, analyses, and interpretations. Our experience in this domain of research has suggested that NaF may be the tool of choice over FDG in assessing atherosclerosis, especially in the setting of coronary artery disease (CAD). Specifically, global NaF assessment appears to be superior in detecting plaques in tissues with high background FDG activity, such as the coronary arteries.

## Introduction

Atherosclerosis is a vascular disease characterized by the formation of plaques and their eventual rupture. The endothelial layer plays a crucial role in the atherosclerotic process. Indeed, endothelial cell dysfunction (ECD) is pivotal in the onset and progression of the events that cause atherosclerosis (Gimbrone and Garcia-Cardena, 2016). ECD occurs via a sequence of signaling events that are part of a complex inflammatory cascade and involves the recruitment of circulating monocytes from the blood into the vessel wall (intima), where they differentiate into macrophages and internalize oxidized low-density lipoproteins to become foam cells. Foam cells are key components of atherosclerotic plaque. Structural remodeling of dead foam cells occurs by their encapsulation by a thin fibrous cap, the rupture of which results in thrombosis and vessel occlusion.

Currently, atherosclerosis and the increasing burden of resulting cardiovascular events constitute a global epidemic (Hansson, 2005; Lozano et al., 2012). Atherosclerosis represents the number one cause of death and disability in the developed world. The characteristic build-up and rupture of arterial plaque is a direct precursor of most cardiovascular and cerebrovascular events, which result in 7.0 and 2.8 million deaths every year, respectively. The economic burden of atherosclerosis is enormous, with annual aggregate inpatient hospital costs of over 10 billion dollars.

The initiation, progression, and complications of atherosclerotic plaque follow slow and gradual sequential events that occur over a lifetime (Mallika et al., 2007; Weber and Noels, 2011). Currently, early identification of at-risk patients poses a challenge for the medical community given the limitations of traditionally utilized assessment tools, such as risk-stratification surveys and mainstay imaging. For example, the Framingham Risk Score quantifies the 10-year risk of developing atherosclerotic disease as a function of risk factors such as dyslipidemia, hypertension, and diabetes, but it offers no means to visualize or predict plaque development. Conventional imaging modalities such as CT and MRI angiography allow physicians to visualize changes that occur in the later stages of atherosclerosis, such as plaque morphology and stenosis, but provide no insight into early detection of plaque formation. Locating and preventing the rupture of “vulnerable” plaques is challenging, as the plaques that are most prone to acute rupture often do not cause clinical symptoms before they rupture (Falk, 1992; Shah, 2003).

Molecular imaging has emerged as an entirely novel means to image and study atherosclerosis very early in the course of disease progression. From a pathophysiological perspective, atherosclerosis is characterized by microscopic processes of inflammation and microcalcification that precede the characteristic plaque buildup in arterial walls detected by traditional assessment methods discussed above. Moreover, the fibrous cap that lies on a lipid-rich necrotic core of oxidized lipoproteins, cholesterol crystals, and cellular debris also comprises calcified deposits. These processes of inflammation and microcalcification are, therefore, prime targets for molecular detection of cardiovascular disease risk.

The two most widely used and studied tracers to detect and characterize atherosclerosis are 18F-sodium fluoride (NaF) and 18F-fluorodeoxyglucose (FDG), which can non-invasively assess microcalcification and arterial inflammation, respectively (Table 1). NaF uptake reflects the exchange of hydroxyl groups of hydroxyapatite crystals for fluoride producing fluorapatite, a key biochemical step in the calcification of atherosclerotic plaque. FDG, an analog of glucose, is used as a surrogate marker for glucose metabolism at the target tissues. FDG is taken up by the cells via transporters and phosphorylated by the hexokinase enzyme inside the cell as FDG-phosphate which cannot be metabolized further. FDG is of particular interest in detecting atherosclerosis due to high metabolic activity of macrophages that reside in plaques and are undergoing hypoxic stress. Furthermore, smooth muscle cells, which have recently been implicated as playing a role in atherogenesis, are highly glycolytic and contribute to visualizing plaques by FDG-PET/CT (Feil et al., 2014; Basatemur et al., 2019; Liu and Gomez, 2019).

**TABLE 1:** Comparison of the utility and limitations between FDG and NaF-PET/CT.

**Clinical utility**		**Limitations**	
**FDG**	**NaF**	**FDG**	**NaF**
Inflammatory processes and infections	Osseous processes due to high bone-to-background ratio	Inaccurate target-to-background measurements in highly metabolic tissues (i.e., heart)	Partial volume effects in small volume bones or intimal plaques
Brain metabolism and MCI/AD	Atherosclerosis, especially in the coronary arteries	Delayed-timepoint imaging may be logistically difficult to achieve	Fewer clinical studies regarding its utility in atherosclerosis
Cancer and neoplasms			

There have been many reviews regarding the importance of FDG-PET/CT and NaF-PET/CT in the diagnosis of atherosclerosis in various settings. In this review, we discuss the role of these two radiotracers in identifying atherosclerotic plaques at the molecular level and before they lead to clinically detectable symptoms. In addition, we examine current published literature and compare the strengths and weaknesses of the existing techniques.

## Molecular Imaging in Atherosclerosis

### Early Attempts to Employ Molecular Imaging Techniques as Correlates to Risk Factors and Prognosis

A study by Yun et al. (2001) represented the first investigation of the association of FDG uptake with atherosclerotic disease. Of 132 patients undergoing whole-body scans, approximately half demonstrated increased FDG uptake in at least one major artery (the abdominal aorta, iliac artery, proximal femoral arteries in whole-body scans and the femoral and popliteal arteries on lower extremity scans), with an increased uptake prevalence in older populations (34% of patients aged 20–40 years, 50% of patients aged 41–60 years, and 61% of patients aged 61–80 years). This result has been replicated in numerous studies in expanded age ranges and in the assessment of additional vasculature. A study by Bural et al. (2008) evaluated FDG uptake in 149 subjects ranging in age from 5 to 83 years. FDG uptake was noted in the major arteries in 145 of the patients, and both prevalence and intensity of FDG uptake in the thoracic aorta, abdominal aorta, iliac arteries, and femoral arteries increased with age. A more recent study by Bural et al. (2016) confirmed this finding. They determined that, in the femoral arteries of 38 patients of varying ages, the uptake ratio of FDG to the adjacent background was significantly increased with age, which suggests that the inflammation associated with atherosclerotic plaques becomes more severe as patients age (Bural et al., 2016). Additionally, Emamzadehfard and colleagues observed an increase FDG uptake in the descending aorta in older (aged 60–75) subjects, when compared to younger (aged 20–30) individuals, in both healthy adults and in those exhibiting cardiovascular risk factors (Emamzadehfard et al., 2017).

While age has been consistently correlated with FDG uptake, association with other conventional risk factors has been less consistent. A further study by Yun et al. (2002) evaluated the frequency of FDG uptake in three major arteries (the abdominal aorta, iliac arteries, and proximal femoral arteries) in 156 patients and its relation to atherogenic risk factors (age, cigarette smoking, hypertension, diabetes, elevated cholesterol, and obesity) and known CAD. Only age and high cholesterol consistently demonstrated a significant correlation with FDG uptake in all three arteries. Additionally, patients with known CAD showed increased FDG uptake in the proximal femoral arteries compared to healthy patients. A study by Strobl et al. (2013) investigated the correlation between FDG uptake in the thoracic aorta, abdominal aorta, common carotid arteries, and iliac arteries with conventional cardiovascular disease risk factors. Different vascular regions demonstrated different correlations. In the thoracic and abdominal aorta, FDG uptake was associated with age greater than 65 and male sex. In the carotid arteries, FDG uptake was associated with age greater than 65, male subjects, and BMI greater than 30. In the iliac arteries, FDG uptake was associated with age greater than 65. Pasha et al. (2015) investigated the correlation between FDG uptake in 76 patients in the aorta and in the peripheral arteries with conventional cardiovascular risk factors. As expected, increased FDG uptake was observed in the peripheral arteries and aorta with increasing age. Cardiovascular risk factors were associated with FDG uptake in the aorta, but not the peripheral arteries. The study by Emamzadehfard et al. (2017) also determined that, in addition to the descending aorta, increased mean FDG uptake was also observed in the aortic arch and ascending aorta in patients with cardiovascular risk factors.

Soon after the early reports appeared in the literature, investigators began exploring the potential role of FDG in detection and characterization of atherosclerosis in various settings. A study by Rudd et al. (2002) investigated FDG uptake in 8 patients who had recently experienced a carotid-territory transient ischemic attack and had severe internal carotid artery stenosis of at least 70 percent. FDG uptake was quantified in plaques in the affected carotid artery and in plaques in the contralateral asymptomatic side. This analysis showed that FDG accumulation was 27% higher in the symptomatic lesions in comparison to the contralateral asymptomatic plaques. Following imaging studies, plaque histology was determined on carotid endarterectomy samples from all 8 patients. Plaque autoradiography demonstrated FDG uptake in macrophage-rich areas of the plaques, notably at the lipid core and fibrous cap border of the lesions, suggesting a potential role for FDG-PET in the assessment of intimal inflammatory response in atherosclerosis.

A study by Paulmier et al. (2008) determined the rate of cardiovascular events in two groups of stable patients with either increased FDG uptake or unremarkable FDG uptake on PET/CT which was performed for cancer staging and follow-up. A calcium index score was calculated for each patient, and subjects from both groups were matched for age, conventional risk factors, and type of cancer. The rates of both remote cardiovascular events (defined as 6 months prior to PET/CT study) and recent cardiovascular events (defined as less than 6 months before or after the PET study) were tracked. In brief, the study revealed that calcium index was the single factor related to remote cardiovascular events, and the extent of FDG uptake was the single factor significantly related to the occurrence of a recent event.

A study by Rominger et al. (2009) evaluated the association of FDG uptake in large arteries of 932 asymptomatic cancer patients with the subsequent occurrence of a vascular event, defined as an ischemic stroke, myocardial infarction, or revascularization. In the 15 of 932 patients (1.6%) who experienced a vascular event, increased FDG uptake was the strongest predictor of said event, more so than conventional risk factors. A study by Marnane et al. (2012) investigated the relationship between FDG uptake and stroke recurrence in 60 patients with a recent stroke, transient ischemic attack, or retinal embolism with ipsilateral carotid plaque stenosis greater than 50%. In 13 of the 60 patients (22%) who suffered from another stroke within 90 days, the FDG uptake in the ipsilateral carotid artery was greater in than those who remained stable. Further analysis in a Cox regression model revealed plaque FDG uptake as one independent predictor of stroke recurrence over conventional risk factors such as age or degree of stenosis. A study by Figueroa et al. (2013) retrospectively identified 513 patients over the age of 30 without a prior history of cardiovascular disease or cancer who had undergone PET/CT. During a follow-up period (median 4.2 years), 44 patients developed cardiovascular disease. Three main findings were observed from the study. First, the degree of FDG uptake and cardiovascular disease incidence were significantly correlated. Second, the inclusion of FDG uptake along with the Framingham Risk Score models of cardiovascular risk improved risk discrimination. Finally, increasing FDG uptake was inversely correlated with the timing of cardiovascular disease defining events.

More recent studies have continued to support the importance of FDG as a molecular indicator of adverse cardiovascular events. Kim et al. (2016) used a cohort of cancer patients to show that arterial FDG uptake, metabolically active malignancy, and the visceral adipose tissue seen on CT scans might have the capacity to predict future ischemic strokes. A study conducted by Iwatsuka et al. (2018) reviewed the medical records of 309 patients age 65 and older who did not have coronary artery disease but had previously undergone FDG-PET/CT imaging to determine the presence of cancer (all patients’ scans were cancer-free). After a median follow-up period of 3.9 years, the authors noted that a high target to background ratio in the arteries was associated with a high likelihood of a coronary heart disease event. Similarly, Tuominen et al. (2019) examined the relationship between pathological FDG uptake and future cardiovascular events in patients who exhibited symptoms of cardiac sarcoidosis. They determined that both total cardiac metabolic activity, as well as right ventricular FDG uptake, were significantly associated with the occurrence of cardiovascular events during a mean follow-up period of 54.7 months following initial FDG-PET scans. Taken together, these recent data demonstrate that FDG-PET has the potential to identify patients who are at risk for cardiovascular events. The data published in the literature indicate that FDG-PET may change the landscape for early identification of patients at-risk for cardiovascular events and may contribute to the development of preventative measures to avoid vascular damage and improve patient outcomes.

Whereas FDG-PET imaging has been shown to be of value in detecting inflammatory processes in the arteries in several studies, NaF has emerged as a superior radiotracer for assessing and quantifying atherosclerosis in the aorta, carotids, and coronary arteries. Derlin et al. (2010) demonstrated in 2010 the feasibility of NaF-PET/CT for detecting vascular microcalcification. The authors performed visual and semi-quantitative methods to quantify NaF uptake. They observed vascular NaF uptake at 254 sites in 57 of the 75 subjects (76%) compared to vascular CT-based calcification at 1,930 sites in 63 patients (84%). Moreover, only 12% of subjects with arterial wall NaF uptake demonstrated corresponding CT calcification, suggesting the high sensitivity of this imaging modality. The same group (Derlin et al., 2011) was able to reproduce their original observation and also demonstrated that common carotid artery NaF uptake is significantly associated with age, male gender, hypertension, and hypercholesterolemia. Morbelli et al. (2014) reached similar conclusions by observing that large vessel NaF uptake in 80 patients with either breast or prostate malignancies (age 65.3 ± 8.2, 20 males) was correlated with Framingham Risk Score and several cardiovascular risk factors including gender, hypertension, smoking, diabetes, and age. Additionally, visible structural changes due to calcified atherosclerosis were not correlated with increased NaF uptake, suggesting that NaF as a molecular calcification marker provides certain specific inflammation about the process.

Blomberg et al. (2017a) demonstrated that thoracic aorta microcalcifications as quantified by NaF-PET/CT revealed a significantly increased risk for cardiovascular disease. This prospective project known as CAMONA determined the role of FDG and NaF imaging in detecting atherosclerosis in 139 subjects, which included healthy controls and individuals at increased risk for fatal cardiovascular disease estimated by the Systematic Coronary Risk Evaluation tool (using gender, age, smoking, systolic blood pressure, and total cholesterol). There was no correlation between FDG uptake and cardiovascular disease risk. The same group also demonstrated that NaF uptake within the coronary arteries is similarly associated with cardiovascular disease risk (Blomberg et al., 2017b). Further analysis of the CAMONA dataset by Arani et al. (2018) and Castro et al. (2017) found a correlation between NaF uptake in the abdominal aorta and common carotid arteries and cardiovascular risk and age. A similar correlation was not found for FDG. Because the carotid arteries are the primary source of blood flow to the brain, NaF-PET/CT may play a major role in detecting atherosclerosis in the vasculature and therefore in the management of patients with cognitive impairment (Borja et al., 2020).

Several recently published studies have demonstrated convincing results by employing NaF as a tracer for detecting molecular calcification, which is indicative of its expanding clinical applications. For example, NaF showed utility in measuring both plaque size and calcification in the arterial beds of transgenic Yucutan minipigs (Nogales et al., 2019). Nogales et al. noted that NaF-PET/CT can reliably detect microcalcifications in the vessels, particularly those in the abdominal aorta, in these animals, suggesting that this methodology can be used to assess plaque burden in entire body. Similarly, Hayrapetian et al. (2019) investigated the utility of NaF-PET in monitoring coronary artery calcifications that are potentially at risk for rupture. They found that NaF uptake can be used as a predictive indicator of the progression of coronary artery atherosclerosis, as well as the degree of coronary stenosis.

### Molecular and Conventional Diagnostic Imaging Modalities

Calcification is a highly specific feature of coronary atherosclerosis. As such, the coronary artery calcium (CAC) score has been widely used as a diagnostic tool for assessing cardiovascular disease risk. However, despite the established association between FDG uptake and conventional cardiovascular risk factors, the relationship between FDG and arterial calcification detected on CT has consistently failed to show any significant correlation. A study by Tatsumi et al. (2003) investigated the relationship between FDG uptake as detected on PET/CT with aortic wall calcification. In a cohort of 85 cancer patients who underwent FDG PET/CT, sites of FDG uptake in the thoracic aortic wall were almost entirely distinct from sites of thoracic aortic wall calcification. FDG uptake was higher in patients older than 65, in those with hyperlipidemia, and in subjects with a prior history of cardiovascular disease.

A study by Ben-Haim et al. (2004) assessed the distribution of both vascular FDG uptake and CT calcifications using PET/CT in 122 patients. A total of 349 vascular sites were identified as abnormal by demonstrating increased FDG uptake, calcification, or both. CT calcification and increased FDG uptake both demonstrated a positive correlation with cardiovascular risk factors and age. CT calcifications were found in 92% of sites and increased FDG uptake was found in 15% of sites, although concomitantly increased FDG uptake and CT calcification was noted in only 7% of sites. Comparable results were described in similarly designed studies by Dunphy et al. (2005), who observed the colocalization between increased FDG uptake and calcification in less than 2% of cases, and by Derlin et al. (2010), who found colocalization in only 12% of cases. A study by Meirelles et al. (2011) examined FDG-PET/CT scans of 100 cancer patients who had undergone at least two PET/CT studies and in whom sites of aortic FDG uptake and calcification were identified. These two image sets (baseline and repeat scans) demonstrated FDG uptake in 70% of the initial scans, which changed in 55% on the second scan. Furthermore, calcification and FDG uptake only overlapped in two cases.

An important study was initiated by Abdelbaky et al. (2013) to demonstrate the relationship between FDG uptake and arterial calcium deposition. They retrospectively identified 137 patients from a single hospital database who had undergone serial PET/CT scans 1–5 years apart. Based on this research, focal FDG uptake was found to be the strongest predictor of future arterial calcification within the same locations after adjusting for risk factors. In other words, localized FDG uptake preceded subsequent calcification over the years. A similar study by Cho et al. (2017) followed 96 patients over 1 year and demonstrated that FDG uptake in the aorta predicted the progression of coronary artery calcification. Enhanced FDG uptake was associated with coronary artery calcification progression in patients without any baseline coronary artery calcification but was not seen in patients with baseline coronary artery calcification.

**FIGURE 1:**
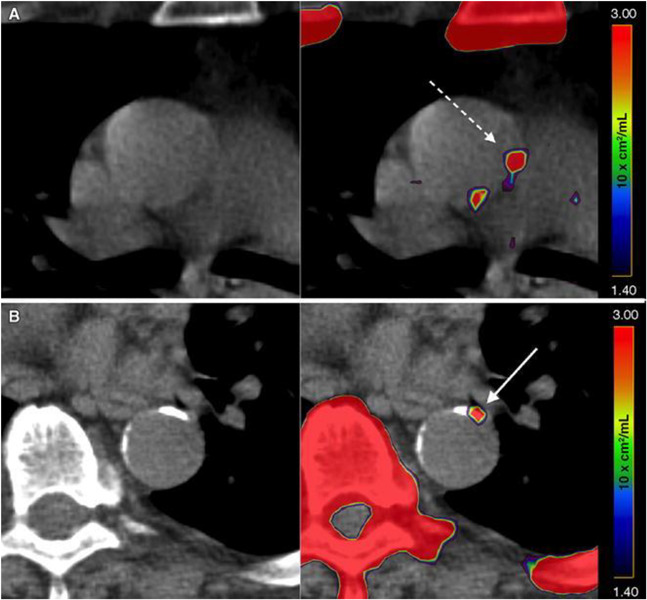
Active and indolent molecular vascular calcification. **(A)** Patient without structural vascular calcification. A positive NaF PET signal, representing molecular vascular calcification, was observed in the ascending aorta (interrupted arrow). **(B)** Patient with active (arrow) and indolent vascular calcification in the descending aorta. Note the intense uptake of NaF in the sternum, vertebra, and ribs (Reproduced from Blomberg et al. (Castro et al., 2017) with permission).

**FIGURE 2:**
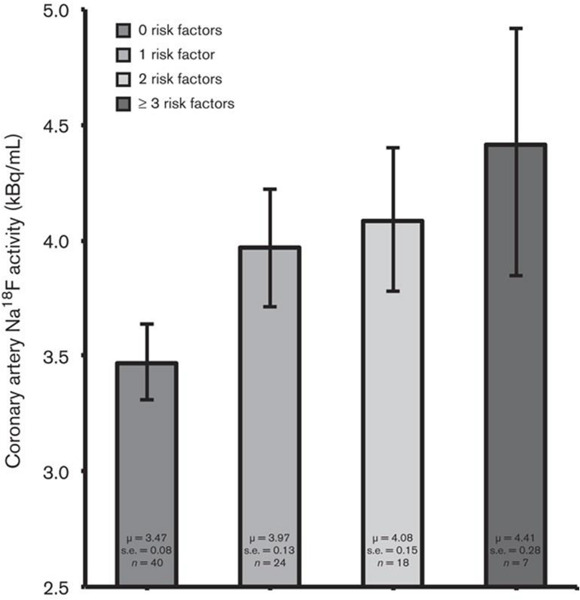
NaF uptake in relation to cardiovascular risk factors. Bar graph showing coronary artery NaF uptake (kBq/ml), adjusted for blood NaF activity, injected NaF dose, and PET/CT technology, in relation to the number of cardiovascular risk factors (i.e., BMI, systolic blood pressure, serum triglyceride concentration, and serum low-density lipoprotein-cholesterol concentration) present. Coronary NaF uptake increased linearly according to the number of risk factors present (*P* < 0.001 for a linear trend). Coronary NaF uptake was significantly lower in the absence of risk factors compared with the presence of one or more risk factors (3.47 vs. 3.97, 4.08, and 4.41 kBq/ml, respectively; *P* < 0.001). Error bars represent the 95% confidence interval of the mean (Reproduced from Blomberg et al. (Figueroa et al., 2013) with permission).

Based on the data that have been published in the literature, it is clear that sites of CT calcification consistently demonstrate no or very minimal uptake of FDG. This indicates that by the time CT calcification is noted, inflammation is no longer an active component of atherosclerotic plaques (McKenney-Drake et al., 2018; Moghbel et al., 2018). The degree of uptake of NaF in the calcified lesions on CT varies considerably (Blomberg et al., 2014b; Figure 1). While some lesions show no uptake, others reveal varying degrees of active molecular calcification (Blomberg et al., 2015b, 2017a,b). These observations indicate that atherosclerotic plaques may be classified as active or inactive based on NaF-PET findings, suggesting the potential clinical utility of NaF uptake in the assessment of atherosclerotic disease burden. Additionally, NaF-PET imaging may be used for the early detection and characterization of patients with atherosclerosis (George, 2012; McKenney-Drake et al., 2018). Animal research studies from our group have validated the reliability of global NaF assessment techniques for early evidence of coronary artery calcification (McKenney-Drake et al., 2018). We have also applied this approach in human studies and have shown significant correlation between NaF uptake and cardiovascular risk factors (Blomberg et al., 2017b; Figure 2). Therefore, we believe NaF-PET will continue to play a major role in assessing atherosclerosis in the major and coronary arteries in the future.

### Investigations Into Plaque Morphology, Histology, and Pharmacotherapy

The early stages of atherosclerosis are characterized by increased endothelial permeability resulting in the buildup of LDL in the arterial intima (van der Wal and Becker, 1999). The exact mechanisms are not completely understood, but subsequent activation of endothelial cells and smooth muscle cells release chemo-attractants, mobilizing monocytes. Monocytes differentiate into macrophages that engulf LDL, transforming into cholesterol-laden apoptotic foam cells, which induce a self-sustaining inflammatory response.

The hallmark of atherosclerosis is plaque formation. “Stable” plaques are unlikely to rupture and are characterized by a thick fibrous cap, a small lipid pool, few inflammatory cells, and a dense extracellular matrix. The continuous cycle of arterial damage and repair in atherosclerosis results in the formation of “vulnerable” plaques (Spacek et al., 2018). In contrast to stable plaques, they are characterized by a thin fibrous cap, a large lipid pool, many inflammatory cells, and few smooth muscle cells. Vulnerable plaques are at an increased risk of rupture. In response, calcification is thought to stabilize vulnerable plaque and reduce the risk of rupture. Initially, apoptotic foam cells are phagocytized by adjacent arterial smooth muscle cells. However, at a certain threshold, apoptotic cells are too numerous to be cleared, giving rise to a necrotic core (Gonzalez and Trigatti, 2017). Vesicles in the necrotic core serve as nucleation sites for microcalcification, which accumulate to form calcifications large enough to be visualized by CT.

The identification of which plaques are “vulnerable” remains a highly desirable goal since the subsequent formation of a thrombus predisposes patients to cardiovascular and cerebrovascular events such as myocardial infarction and stroke. As a marker of inflammation, FDG represents a key component of the atherosclerotic disease process. It is relatively well established that FDG uptake in the setting of atherosclerosis is related to the inflammatory process of the activated macrophages within plaques.

A study by Tawakol et al. (2006) examined 17 patients with severe carotid artery stenosis who underwent PET/CT prior to endarterectomy. Following surgery, immunohistochemistry was performed on the excised plaque extracts. The study specifically examined the association of FDG uptake with CD68 level, serving as a measure of macrophage content, and demonstrated a strong correlation between these two variables. A similar study by Myers et al. (2012) resulted in significantly different results. The study investigated the correlation between FDG uptake in the aorta, carotid arteries, and femoral arteries in 30 patients who underwent an atherectomy procedure for symptomatic common or superficial femoral arterial disease. After the procedure, immunohistochemistry was performed on the excised plaque extracts to quantify CD68 uptake, once again serving as a measure of macrophage content. In 34 plaque specimens obtained, no correlation was noted between FDG uptake and CD68 level. Finally, a study by Figueroa et al. (2012) designed a similar study but used investigators blinded to the clinical, PET, and histological data to characterize plaque morphology as a risk factor in 34 patients scheduled for endarterectomy, 10 of whom had plaque histologically assessed for CD68 staining. Both FDG uptake and CD68 staining were higher in vascular segments that contained plaque and in plaques characterized as possessing high-risk morphology.

A more recent study by van der Valk et al. (2016) investigated the relationship between FDG uptake and atherogenic lipoprotein(a), a proinflammatory oxidized phospholipid transporter associated with accelerated arterial inflammation. In 30 subjects with elevated lipoprotein(a) levels, FDG uptake was significantly elevated compared to 30 control subjects with normal lipoprotein(a) levels.

Other studies have taken advantage of animal models to investigate the biological characteristics of atherosclerotic plaques. Ogawa et al. (2004) studied FDG uptake in an animal model of atherosclerosis, Watanabe heritable hyperlipidemic (WHHL) rabbits. Eleven WHHL and 3 control rabbits were injected with FDG and imaged with PET/CT, followed by the removal of both the thoracic and abdominal aortas 4 h later. Histologic analysis was performed to quantify macrophage content. In the control rabbits, the aortas were devoid of plaque and no macrophages were seen on histological examination. In the WHHL rabbits, a thick intima representing plaque content as well as significant macrophage content were observed. FDG uptake in the WHHL rabbits was strongly correlated with atherosclerotic lesions and macrophage content. Of note, FDG may have the potential to be used in drug development studies. As indicated by Plana et al. (2018), FDG-PET imaging can identify patients at risk for doxorubicin-mediated cardiotoxicity following chemotherapy treatments, though this has only been determined in mice models and has not yet been widely clinically tested.

Joshi et al. (2014) compared coronary NaF uptake in areas of culprit and non-culprit plaques in 40 patients with myocardial infarction and compared NaF uptake to intravascular ultrasound examination in 40 patients with stable angina. In patients with myocardial infarction, culprit plaques and sites of atherosclerotic plaque ruptures demonstrated high focal NaF uptake. Moreover, histological examinations of focal regions of uptake showed active calcification and necrotic changes. On the other hand, patients with stable angina who demonstrated high NaF uptake also demonstrated high-risk characteristics on ultrasound, including vascular remodeling and calcification. A more recent study by Kim et al. (2019) performed FDG- and NaF-PET/CT imaging in 20 stroke patients with culprit and non-culprit carotid plaques. High radiotracer uptake was present in both groups, and the target-to-blood ratio demonstrated a significant increase in uptake in culprit atheromas. Vesey et al. (2017) used both FDG and NaF-PET/CT to determine the superior modality in identifying carotid plaques in 26 patients with recent ischemic strokes. They determined that, while both NaF and FDG uptake were significantly associated with predicted cardiovascular risk, only NaF was significantly correlated with plaque burden and associated plaque characteristics. Vesey et al. also used histologic and micro PET/CT to confirm the potential for NaF to be used as a tracer in the identification of microcalcifications. Similarly, Irkle et al. (2015) used sequential staining of carotid artery plaques to identify the specificity of binding of NaF to regions of carotid artery plaques. Using Alizarin Red, a calcification marker, along with macrophage and endothelial cell neovascularization markers, they determined that the NaF radioactivity signal was only significantly associated with the Alizarin Red stain, which is reflective of the ability of NaF to identify calcifications in the carotid artery and not the presence of infection or other related cellular processes. Taken together, these recent data suggest that NaF is a powerful indicator of the presence of microcalcifications, which may potentiate its role in the surveillance of plaque development in the vasculature.

A study by Beheshti et al. (2011) examined NaF-PET/CT images in 51 to determine cardiac and aortic uptake. The authors correlated NaF uptake to age, suggesting the potential for NaF in the assessment of atherosclerosis. Additionally, this study was the first to utilize global disease assessment with NaF-PET. Global disease assessment was first described by Alavi et al. (1993) by measuring FDG uptake in the brains of patients with mild cognitive impairment or Alzheimer’s Disease. Recently, Sorci et al. (2019) adopted this approach in their 2019 study which compared NaF-PET/CT and Framingham Risk Score to CAD risk factors, including age and BMI, in 86 controls and 50 CAD patients. The authors observed that both NaF-PET/CT and Framingham Risk Score were increased in CAD patients. Moreover, NaF-PET/CT but not Framingham Risk Score could differentiate between patients and controls, and global NaF uptake was significantly correlated with both age and BMI. Taken together, these studies all suggest a major role for global NaF assessment in individuals at risk for atherosclerosis and CAD.

McKenney-Drake et al. (2018) demonstrate that early identification of vascular microcalcification by NaF-PET/CT has significant prognostic value. Coronary NaF uptake and histopathological and sonographic assessment were compared between 13 lean (control) Ossabaw swine and 11 animals with metabolic syndrome and early CAD. The researchers found that the animals with metabolic syndrome demonstrated both 2.5-times higher coronary vascular uptake and 100-times higher plaque burden as the control animals. Furthermore, a recent publication by Kwiecinski et al. (2011) investigated 293 patients with CAD to determine if NaF-PET/CT could be used as a predictor of future myocardial infarction. They found that only patients who experienced a myocardial infarction during the 42-month follow-up period demonstrated increased NaF uptake in the coronary arteries in the initial scans. Importantly, the authors concluded that NaF uptake in the coronary arteries could be used as an independent prognostic factor for myocardial infarction occurrence. These data suggest the prognostic potential of NaF PET/CT, in correlation with histological and pathological changes of atherosclerotic calcification in CAD.

FDG-PET/CT have been used with varying success to investigate the efficacy of potential atherosclerosis therapeutics. Kim et al. (2018) found that FDG-PET could not be used to evaluate statin efficacy. In their prospective study of 13 acute coronary symptom patients imaged before and after 1 month of 20 mg/day atorvastatin, they found no difference in FDG uptake within the carotid arteries. On the other hand, Choo et al. (2018) demonstrated in 2018 that atorvastatin and pioglitazone co-therapy is associated with decreased vessel inflammation as assessed by FDG-PET/CT relative to atorvastatin alone in 41 patients with coronary artery disease. This data suggest that FDG-PET should be applied toward therapies that alter vascular inflammation, such as tumor necrosis factor-alpha modulators or interleukin-6 blockers, rather than plaque development. Based on the limited utility of FDG-PET/CT in this domain, further studies should utilize NaF-PET over FDG to assess atherosclerotic disease and consequent response to treatment. For example, Moss et al. (2019) used NaF-PET to examine the effects of ticagrelor, a platelet adenosine diphosphate P2Y12 receptor antagonist, on troponin I levels in patients with multivessel coronary artery disease. Using NaF uptake in the coronary arteries to monitor the presence of plaques, the authors confirmed that ticagrelor did not significantly influence troponin I levels in patients with multivessel coronary artery disease, which suggests that this factor is not a reliable marker for monitoring antagonism to the P2Y12 receptor on platelets. This study demonstrates a potential role for NaF in pharmaceutical development, which may be of use to physicians in future clinical trials.

### Limitations of Molecular Imaging in Assessing Atherosclerosis

A major limitation of FDG as a means to investigate atherosclerosis has been its non-specific uptake by myocardial tissue as well as the arterial wall. This is particularly of major concern in the coronary arteries, since spillover from the physiologic activity of the heart obscures detection of atherosclerotic inflammation (McKenney-Drake et al., 2018). A study by Williams and Kolodny (2008) proposed a method of circumventing this limitation in certain settings. In brief, myocardial glucose metabolism and undesired FDG uptake is suppressed by requiring subjects to consume a very high-fat, low-carbohydrate, protein-permitted diet (VHFLCPP) 3–6 h prior to image acquisition. In comparison to a fasting control group, the average FDG uptake in the VHFLCPP group was suppressed. The utility of the VHFLCPP diet protocol was replicated by Wykrzykowska et al. (2009), who demonstrated “good” or “adequate” myocardial suppression in 20 out of 32 patients. The cases of inadequate suppression were due to self-reported dietary non-adherence. Other than myocardial spillover, FDG uptake is also limited by its spatial resolution, as visualization of uptake in the millimeter range is past the physical limits of PET imaging (Bural et al., 2006).

**FIGURE 3:**
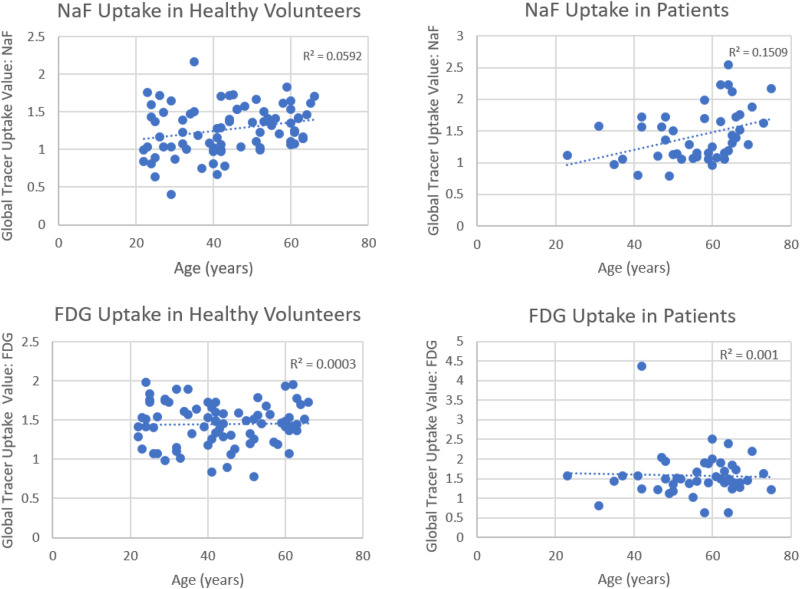
Correlations between global tracer uptake values (GTUV) of NaF, FDG and age. Significant correlations were present for NaF uptake in both the healthy volunteers and patients (upper panels) but not for F-FDG in these subject groups (lower panels) (Reproduced from Arani et al. (Blomberg et al., 2015b) with permission).

**FIGURE 4:**
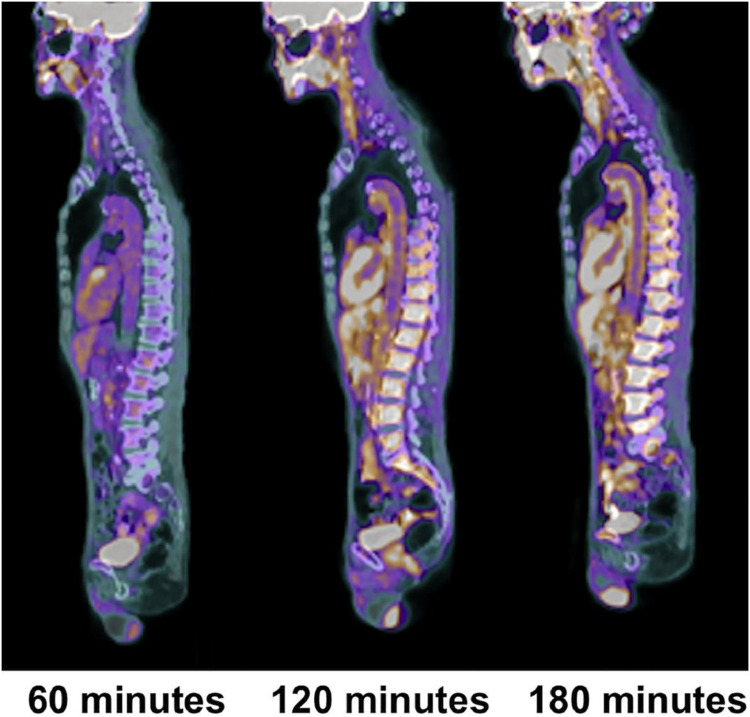
Changes in aortic wall and luminal blood FDG activity at different imaging time-points as seen on sagittal FDG PET images of the thoracic aorta. With time, luminal blood activity decreases while the aortic wall activity increases, which improves the arterial wall-to-blood contrast (superior target-to-background ratio) (Reproduced from Moghbel et al. (Gonzalez and Trigatti, 2017)with permission).

**FIGURE 5:**
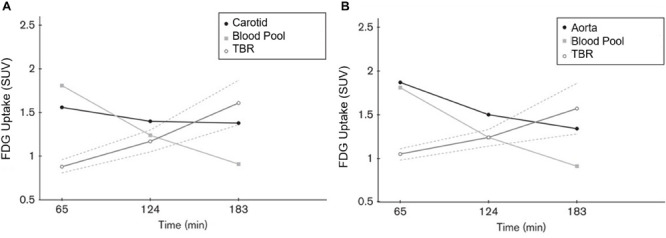
Graph plotting the FDG activity (SUV) in the carotid arteries **(A)**, aorta **(B)** and the right atrial blood pool at 65, 124, and 183 min after FDG administration. The ratio between the arterial and blood pool activity increased with time. The interrupted lines represent the 95% confidence interval of TBR values. We would like to emphasize the TBR measurements differ at 1, 2, and 3 h. This indicates the lack of reliability of TBR for accurate quantification of uptake in the atherosclerotic plaques (Reproduced from Blomberg et al. (van der Wal and Becker, 1999) with permission).

Based on recent publications in the literature, it is apparent that FDG-PET possesses low sensitivity and specificity as a molecular probe for detecting inflammation in atherosclerotic plaques (McKenney-Drake et al., 2018; Moghbel et al., 2018; Arani et al., 2019; Figure 3). This is primarily because FDG is not only taken up by inflammatory cells, but also by other cellular structures in the vessel wall. In other words, smooth muscle cells in the arterial wall, which are known to be highly glycolytic, may have high levels of FDG uptake and this decreases the specificity of FDG-PET imaging for detection of atherosclerotic plaques. Furthermore, the suboptimal spatial resolution of PET (in the range of 5–8 mm) results in substantial underestimation of the degree of uptake in the plaques. Efforts have been made to correct for partial volume effects to accurately quantify the degree of inflammation in plaques (Blomberg et al., 2015a). This would require administering contrast agents along with PET, which is not routinely employed for the assessment of cardiovascular imaging with PET/CT (Blomberg et al., 2015a). Furthermore, the majority of reports in the literature regarding the detection of inflammation in atherosclerotic plaque is based on FDG-PET imaging only 60 min following administration of the tracer. The degree of clearance of FDG is relatively slow, and substantially high levels of the tracer that remain in the circulation affect the accuracy of measurements made with this approach (Cheng et al., 2013). In other words, spillover from the high levels of intravascular tracer activity results in an overestimation of inflammatory reaction in plaques. Efforts have been made to minimize this undesirable effect on optimal quantification of atherosclerosis by imaging at delayed time points of up to 3 h (Blomberg et al., 2013, 2014a, 2015a; Moghbel et al., 2018; Figure 4). Therefore, the results from existing literature that are based on 60-min imaging are somewhat inaccurate and need to be verified by optimally delayed imaging protocols. We should also emphasize that target-to-background ratio, which is commonly used for assessing atherosclerosis, is of questionable value for overcoming blood pool activity effect on accurate quantification of atherosclerosis (Figure 5). Based on studies that have been performed at 1, 2, and 3 h post-injection and TBR quantification, it is clear that target-to-background ratio provides unreliable numbers for the presence and degree of inflammation in plaques (Blomberg et al., 2013, 2015a). Finally, a number of FDG-PET/CT studies have been performed in patients with numerous co-morbidities, such as cancer, subsequent chemotherapy/radiation therapy, and other inflammatory conditions. Upregulation of proinflammatory cytokines due to these abnormalities may therefore, influence FDG uptake in other structures, similarly suggesting that NaF-PET/CT may be the tool of choice for the clinical assessment for atherogenic microcalcifications.

The clinical utility of NaF in the assessment of atherosclerosis is still being evaluated. While microcalcifications may be detected by NaF-PET/CT and are believed to precede macroscopic plaque buildup in arterial walls, the mechanistic relationship of microcalcifications toward the overall pathobiology of atherosclerosis has yet to be elucidated. Additionally, the small size of atherosclerotic plaques is comparable to the typical spatial resolution of PET, leading to potential inaccuracies in NaF quantification due to partial volume effects. Recently, Cal-Gonzalez et al. (2018) developed partial volume correction techniques to improve NaF-PET quantification of atherosclerotic plaques to help mitigate this issue. Also, global assessment of NaF uptake in the coronaries and major arteries may overcome this limitation. Uptake of NaF in the bony structures may adversely affect the results in the arteries adjacent to the skeleton. We believe that further translational and clinical research using NaF-PET/CT will answer these questions and provide a more definitive review for the role of this imaging modality.

PET-based imaging modalities may further aid in the assessment of atherosclerosis and cardiac disease. Specifically, radiolabeled nanoparticles may be used to assess macrophage activity (de Barros et al., 2012). de Barros et al. (2014) utilized dextran-coated iron oxide nanoparticles to assess the macrophage burden in the coronary arteries of apolipoprotein-E-deficient mice. They found the ApoE knockout mice demonstrated high cardiac uptake of radiotracer than controls. Additionally, among ApoE knockout mice, radiotracer was specifically localized to diseased hearts but not healthy hearts. This approach may allow detection of inflammation in the coronary arteries which is unachievable with FDG. Additionally, many studies have been performed using tracers other than NaF and FDG which have shown success in the study of cardiovascular pathology. For example, Thackeray et al. (2018) examined the effects of myocardial infarction on inflammation in the heart and brain in mice models using mitochondrial translocator protein (TSPO) as their PET ligand. They determined that TPSO uptake was significantly increased in the infarct region 1 week following infarction, as well as in remote cardiomyocytes 8 weeks later, correlating with left ventricular remodeling and heart failure. In the brain, TSPO uptake was increased in the microglia, which reflects the presence of neuroinflammation occurring alongside the infarction. This suggests that using TSPO as a PET ligand may be of interest in future large-scale studies, as it has the potential to identify multiple areas of cellular and tissue damage. Additionally, Tarkin et al. (2019) used 68Ga-DOTATATE, a somatostatin receptor subtype-2 PET ligand, alongside FDG to examine residual myocardial inflammation following myocardial infarction. They determined that, not only was 68Ga-DOTATATE uptake significantly correlated with post-infarction myocardial inflammation, but it also had less background signal than the FDG scans, which may point to its superiority over FDG as a PET tracer.

Both NaF and FDG have been used as proxies for the detection of early stage CVD. We may see refinement in the specific processes captured by each radiotracer, and how exactly they relate to large-scale pathology. Given that NaF is an effective tool for the detection of microcalcification, and that FDG is only proven as an inflammatory marker, more research with standardized protocols and methods is needed to assess FDG’s utility as a diagnostic tool for cardiovascular illness. A literature search by Huet et al. (2015) identified 46 different quantification methods for the quantification of FDG uptake in atherosclerotic plaques used in 49 studies. Since inflammation is a critical risk factor for plaque rupture, likely, there is still potential for this radiotracer in the area of cardiovascular health, which will be better understood as its relationship with CVD and acute cardiac events is increasingly quantified. A recent example of the potential benefits of using NaF and FDG concurrently is demonstrated by recent data from Chowdhury et al. (2019), which sought to predict the development of restenosis following percutaneous transluminal angioplasty in individuals with peripheral artery disease. The authors identified significantly increased femoral artery inflammation using FDG, as well as a significant increase in the degree of microcalcification present using NaF, in patients with restenosis as compared to individuals without restenosis. This study demonstrates one of many possibilities in the future of using NaF and FDG together, which will help clinicians evaluate diseases with complex presentations involving both microcalcification and inflammation.

### Future Directions in PET Imaging

Based on the large volume of data that have been introduced to the literature and discussed in this review, it is clear that PET imaging will have a major role to play in assessing atherosclerosis in the major and coronary arteries. Recent evidence demonstrates the feasibility of NaF in assessing atherosclerosis in the aorta, carotid arteries, and coronary arteries. This agent is rapidly cleared from the circulation and therefore the degree of background activity, which is a major source of error for such measurements, no longer interferes with accurate quantification of atherosclerotic plaques. In other words, significant clearance of NaF by the bony skeleton and the kidneys over a short period of time results in very minimal activity remaining in the blood even after 1 h. Therefore, the need for target-to-background ratio measurements is obviated by using NaF-PET. Furthermore, the specificity of microcalcification to the atherosclerotic plaques allows measurements of CAD which is almost impossible to achieve by FDG. Since coronary artery atherosclerosis is a serious and potentially fatal predisposing risk factor in patients with CAD, NaF imaging has great promise for becoming the study of choice for early detection and accurate quantification of the disease process. We believe NaF-PET imaging will obviate the need for CT calcification assessment which reveals irreversible disease process in the coronary arteries and therefore is of limited value in the management of these patients. Large multi-center trials are necessary to further confirm the role of PET in this disease (McKenney-Drake et al., 2018; Moghbel et al., 2018; Hoilund-Carlsen et al., 2019).

## Author Contributions

All authors made a substantial contribution to this review, and approved it for publication.

## Conflict of Interest

The authors declare that the research was conducted in the absence of any commercial or financial relationships that could be construed as a potential conflict of interest.
